# Supplementary material for: Integration of rate and phase codes by hippocampal cell-assemblies supports flexible encoding of spatiotemporal context
Source: Nat Commun. 2024 Oct 22;15:8880. doi: 10.1038/s41467-024-52988-x (PMC11496817; doi:10.1038/s41467-024-52988-x)
Supplement: Supplementary file 1 — Supplementary Information [file 41467_2024_52988_MOESM1_ESM.pdf]

Supplementary Information for

Integration of rate and phase codes by hippocampal cell-assemblies supports flexible encoding of spatiotemporal context

Eleonora Russo[1,2,3,✉], Nadine Becker[4,5], Aleks P. F. Domanski[4], Timothy Howe[4], Kipp Freud[6], Daniel Durstewitz[2,7], Matthew W. Jones[4,7]

[1] The BioRobotics Institute, Department of Excellence in Robotics and AI, Scuola Superiore Sant'Anna, 56127 Pisa, Italy.
[2] Dept. of Theoretical Neuroscience, Central Institute of Mental Health, Medical Faculty Mannheim, Heidelberg University, 68159 Mannheim, Germany.
[3] Department of Psychiatry and Psychotherapy, University Medical Center, Johannes Gutenberg University, 55131 Mainz, Germany.
[4] School of Physiology, Pharmacology & Neuroscience, Faculty of Life Sciences, University of Bristol, University Walk, Bristol BS8 1TD, UK.
[5] Current address: Nanion Technologies GmbH, Ganghoferstr. 70A, D-80339 Munich – Germany.
[6] School of Computer Science, Merchant Venturers Building, University of Bristol, Woodland Road, Bristol, BS8 1UB, UK.
[7] Equal contribution.
✉ Corresponding author, email: eleonora.russo@santannapisa.it

Supplementary figures

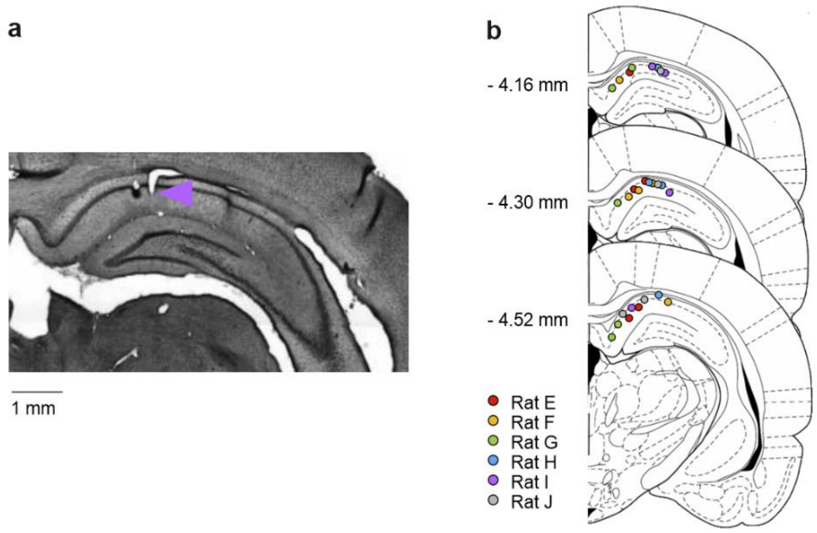

Supplementary Fig. 1 | Histology. (a) Coronal section showing histological confirmation of the tetrode placement (lesion site marked with arrowhead) in the CA1 of one rat; (b) Dorsal CA1 tetrode placement for all recorded rats.

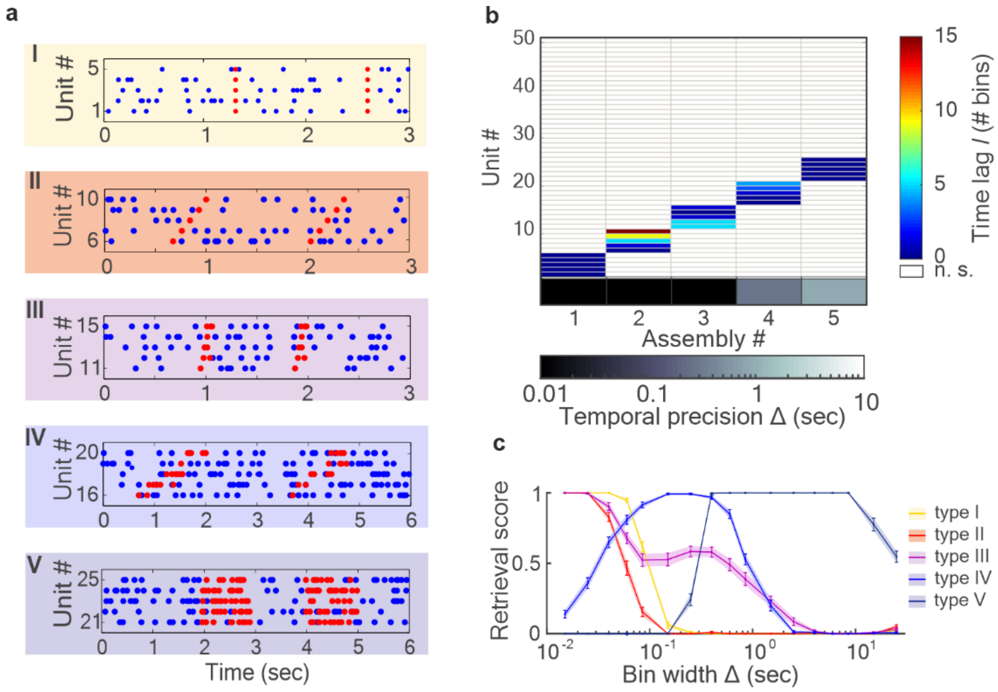

Supplementary Fig. 2 | Cell assembly detection with CAD. Hippocampal assemblies were detected by applying the method for Cell Assembly Detection (CAD) presented in [1]. CAD can detect assemblies in an unsupervised fashion returning for each assembly the identity of its composing units, the activation pattern, and the temporal resolution of the unit coordination. To exemplify the information returned by CAD, we here report the algorithm performance when applied to 50 simulated units containing 5 distinct assemblies of 5 units each. To showcase the wide range of CAD sensitivity, the simulated assemblies differed both in activation pattern and temporal resolution. (a) Rasterplots of 25 of the 50 simulated units (in blue), showing the activation of the five simulated assemblies (in red): type I – highly precise lag-0 synchronization; type II – highly precise sequential pattern; type III – highly precise spike-time pattern without clear sequential structure; type IV – rate pattern with sequential pattern; V – synchronous rate increase. Units from 26 to 50 were not included in any assembly. (b) Assembly-assignment matrix showing the output of CAD. Each detected assembly corresponds to a column of the assembly-assignment matrix. Colored units (rows) have been identified as part of the assembly. The color indicates the lag between the activation of the unit and that of the first unit active within the assembly. Along the abscissa, in grey-scale, the temporal resolution of the detected assembly. (c) Fraction of correctly assigned units (retrieval score) as a function of the temporal resolution (bin size) at which the algorithm scans the spike train. Data averaged across 70 independent runs, error bars = SEM. Thanks to the non-stationarity correction implemented in the algorithm (see [1]), CAD detects assemblies only at their characteristic temporal resolution. Thus, precise assemblies of type I and II were only detected at very small bin sizes, while rate assemblies of type IV and V only at bigger bin sizes. Interestingly, the type III assembly was detected both at small and larger bin sizes because of the precise timing in the relative activation of assembly units, and the change in firing rate produced by its extended activation pattern. Figure taken from ref. [1] under CC-BY 4.0 license.

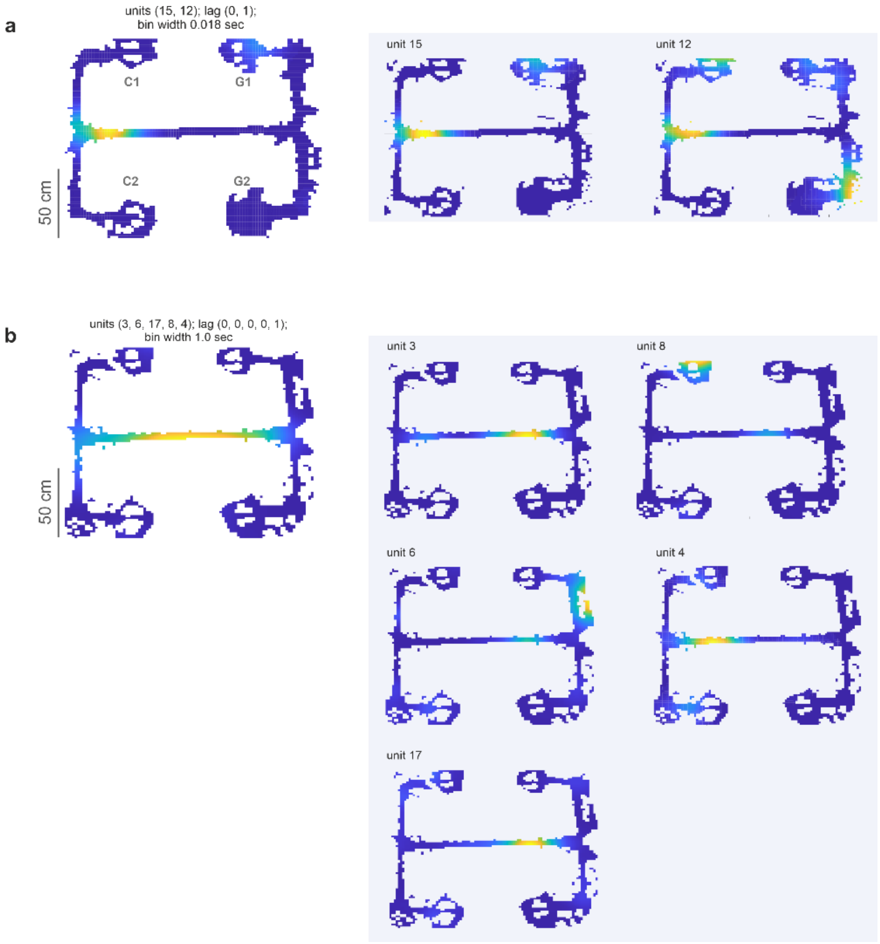

Supplementary Fig. 3 | Activation maps of assemblies and their composing units. Example of activation maps of a spike- (a) and a rate- (b) assembly and of its composing units. While single units fired in multiple place fields throughout the maze, assembly activations were more selective.

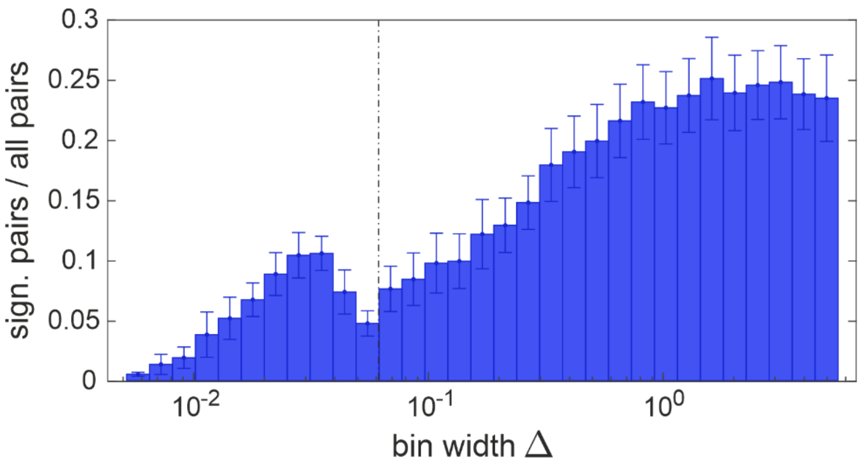

Supplementary Fig. 4 | Temporal precision of CA1 assemblies detected outside of SWR epochs. Same as in Fig. 1b, but with assemblies detected on spike trains where epochs with SWR were excluded. Bars show weighted mean and SE computed across 6 animals and 4 sessions (sessions without assemblies were excluded, *n. sessions* = 23, *n. assembly pairs* = 4826). Source data are provided as a Source Data file.

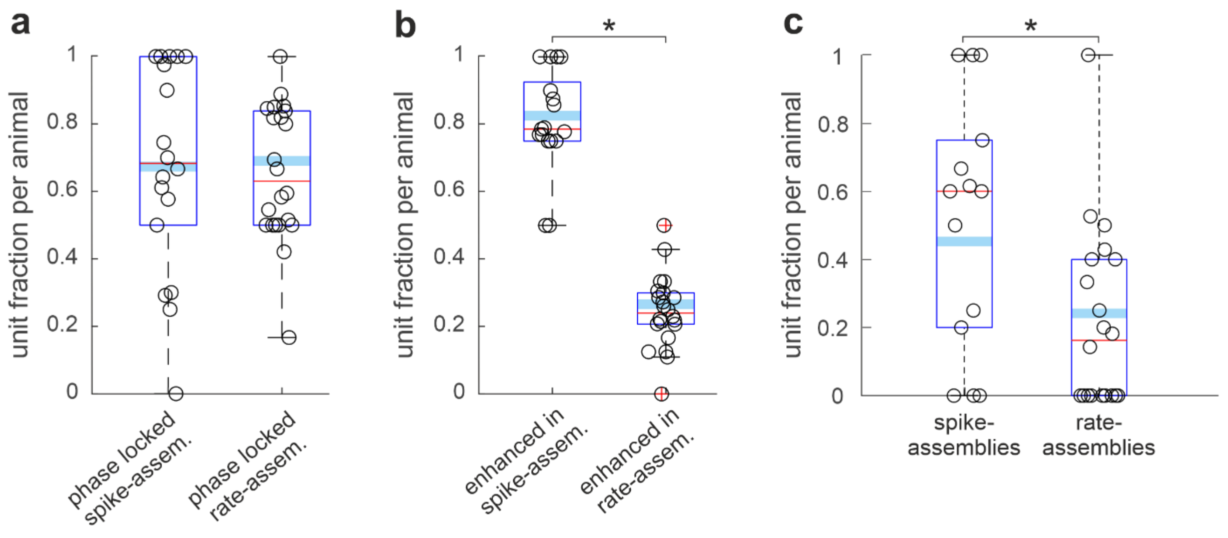

Supplementary Fig. 5 | Theta-phase modulation of assemblies detected outside of SWR epochs. (a) Same as Fig. 2b, (b) same as Fig. 2c, (c) same as Fig. 3b but computed on assemblies detected excluding epochs with SWR. In all three panels we tested significance with generalized linear mixed-effects models to account for rat identity and recording session as covariates. The models and their output statistics are (a) generalized linear mixed-effects model of the probability of a unit to phase-lock when firing within an assembly according to the assembly type, spike- vs rate-assembly: F(1,1812) = 1.9, p = 0.17; (b) generalized linear mixed-effects model of the probability of a unit to increase phase modulation when firing within an assembly according to the assembly type, spike- vs rate-assembly: F(1,1301) = 178.4, $p = 3.2\ 10^{-38}$; (c) generalized linear mixed-effects model of the probability of a unit to phase-shift when firing in different assemblies according to the assembly type, spike- vs rate-assembly: F(1,230) = 6.93, $p = 9.0\ 10^{-3}$. Data from 4 sessions of 6 rats, sessions where no units met the inclusion criteria were excluded (n = 18, 22 in (a), n = 17, 22 in (b), n = 14, 20 in (c)). Source data are provided as a Source Data file.

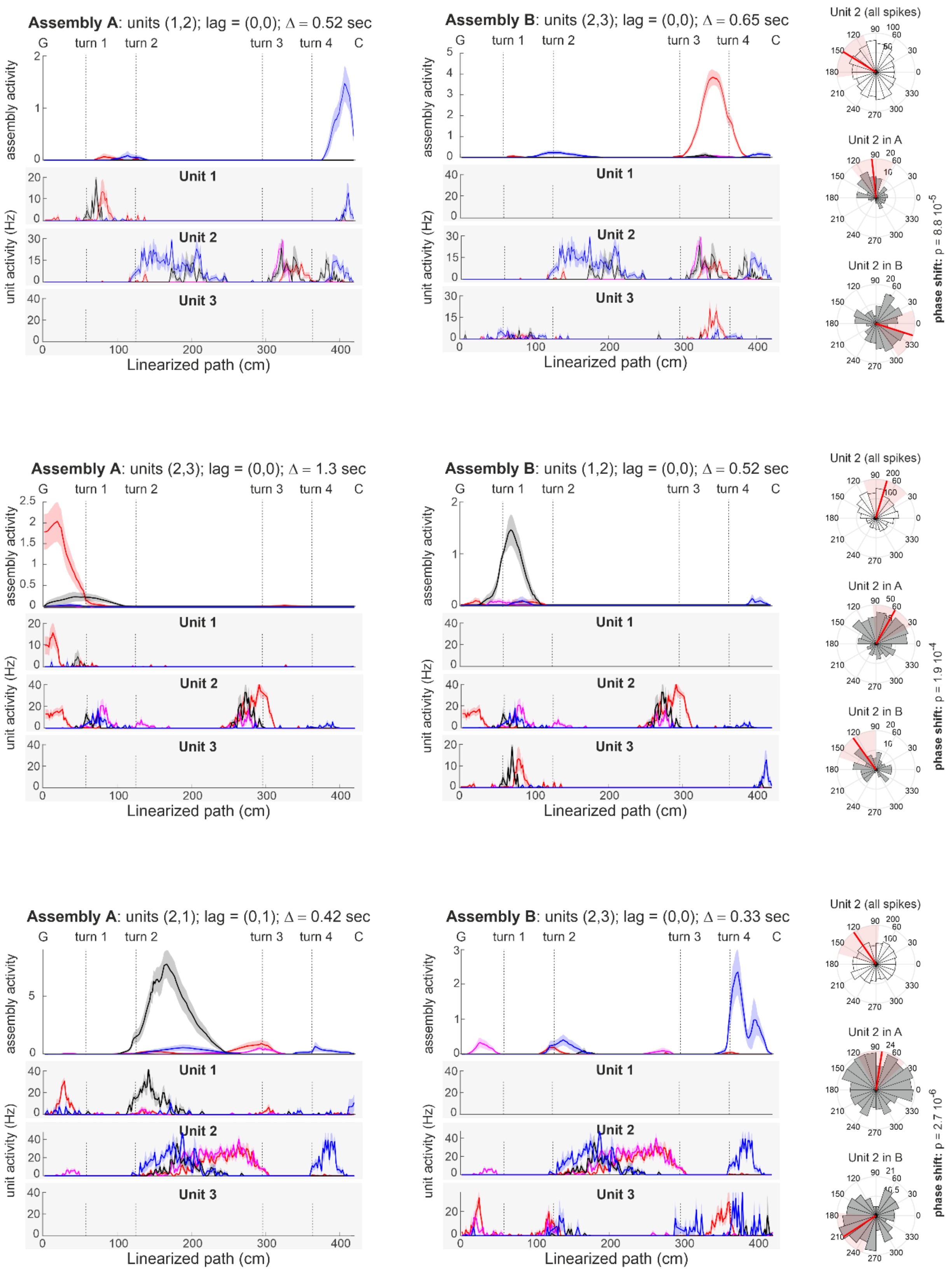

Supplementary Fig. 6 | Changes in unit information coding when joining different assemblies. Same as **Fig. 3c**, three additional examples of units with changing phase preference when active in different assemblies.

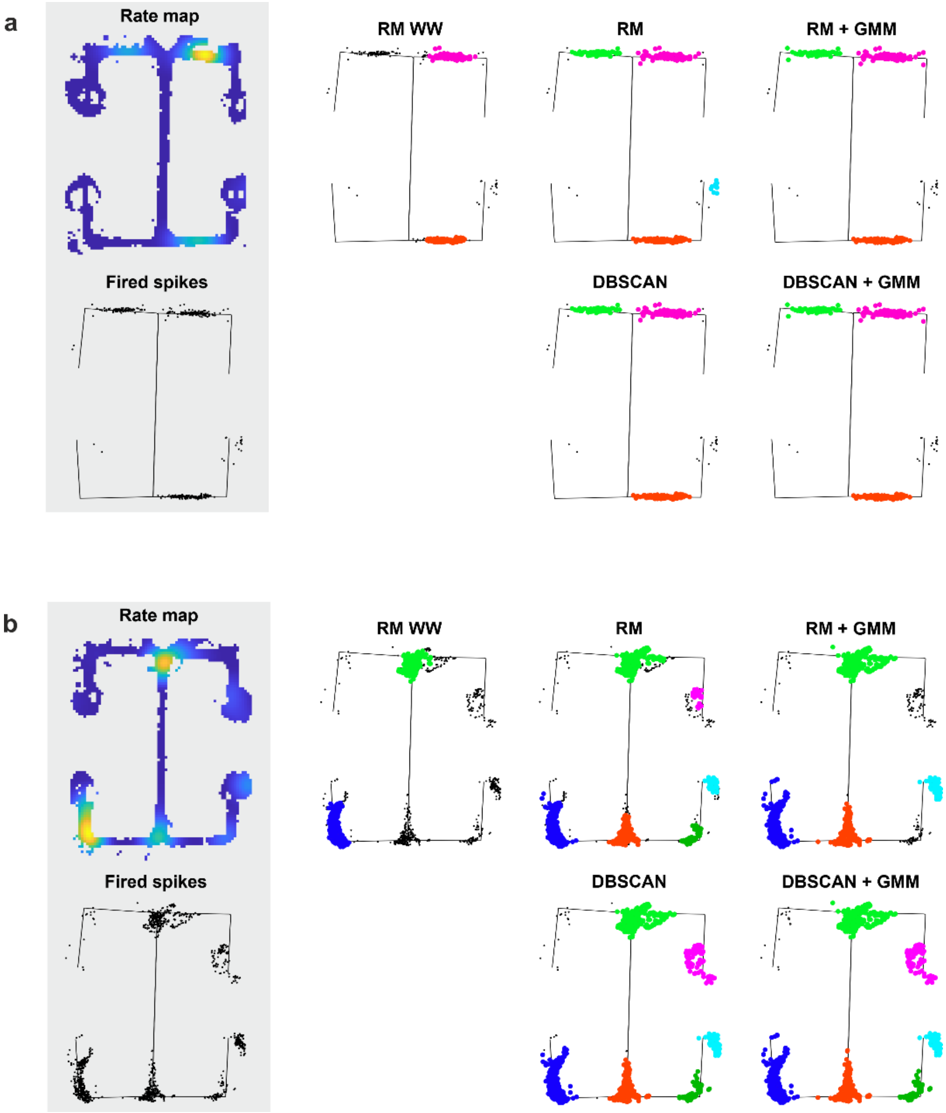

Supplementary Fig. 7 | Method comparison for place field detection. We compared the output of 5 different place field detection methods: a thresholded rate map based detection algorithm presented in Wirtshafter and Wilson [2] (**RM WW**); a rate map based detection algorithm adapted from Wirtshafter and Wilson (**RM**); the RM algorithm combined with a Gaussian mixture model (**RM + GMM**); a density-based spatial clustering algorithm (**DBSCAN**); the DBSCAN algorithm combined with a Gaussian mixture model (**DBSCAN + GMM**) (see Methods for discussion and description of the place field detection algorithms). In (a) and (b) in the grey panel the rate map (top) and the position on the maze (bottom) of the recorded spikes of two example place cells. On the right, spike's positions color-coded according to the place fields detected by the 5 different algorithms. Different colors corresponded to different place fields. As visible in the two examples, in units with multiple place fields, thresholding the rate map at one standard deviation above the average firing rate of the unit might result in the failure to detect those fields where the unit consistently becomes active at lower frequencies.

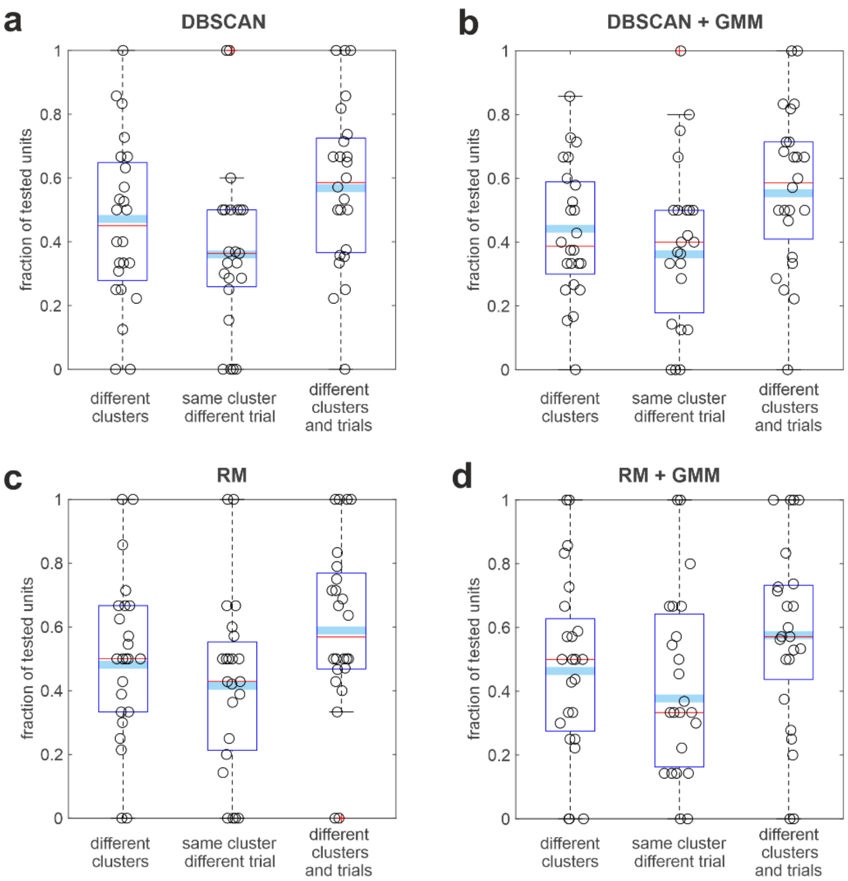

Supplementary Fig. 8 | Fraction of units changing preferred theta-phase for different place fields and trial types. To probe the robustness of the results reported in Fig. 5b we performed the same analysis on sets of place fields identified with alternative techniques. From panel (a) to (d) we used place fields detected respectively by: a density-based spatial clustering algorithm (**DBSCAN**); the DBSCAN algorithm combined with a Gaussian mixture model (**DBSCAN + GMM**); thresholding the unit rate map (**RM**); the RM algorithm combined with a Gaussian mixture model (**RM + GMM**) (see Methods for discussion and description of the place field detection algorithms). Panel (b) is the same as Fig. 5b and reported here for comparison. Data from 4 sessions of 6 rats, sessions where no units met the inclusion criteria were excluded (*n* = 24, 23, 24 in (a), *n* = 24, 23, 24 in (b), *n* = 24, 23, 24 in (c), *n* = 24, 23, 24 in (d)). Source data are provided as a Source Data file.

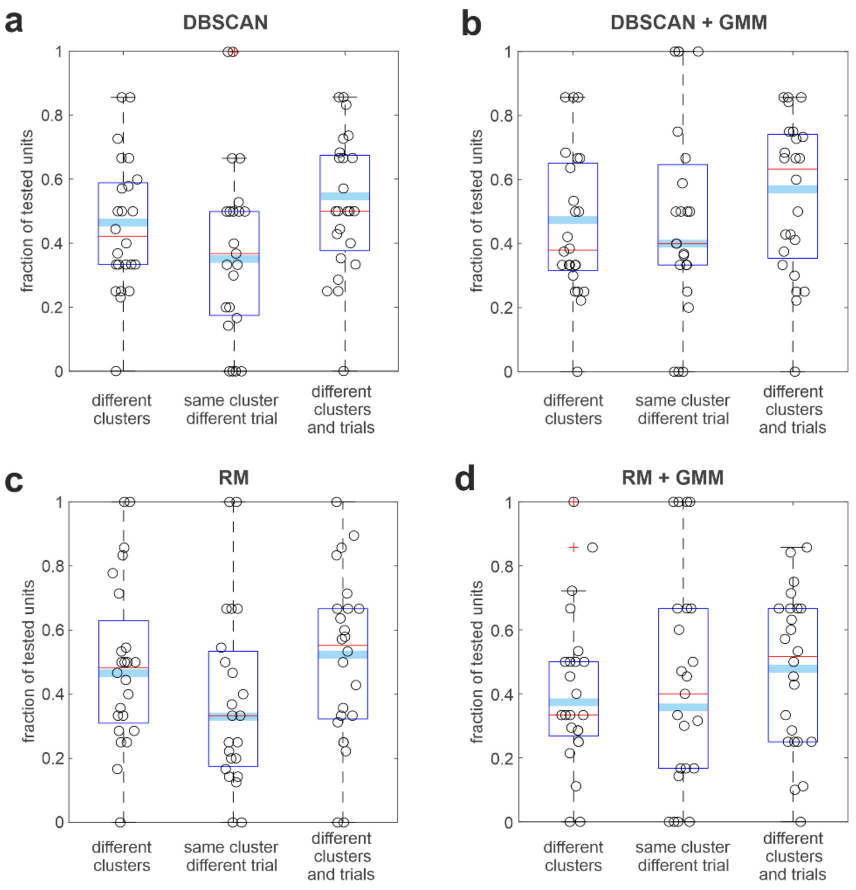

Supplementary Fig. 9 | Fraction of units changing preferred theta-phase for different place fields and trial types (only high theta power and no SWR included). Same as Supplementary Fig. 8 but with analyses performed on spikes fired in epochs of high theta-power and outside of SWR epochs. Data from 4 sessions of 6 rats, sessions where no units met the inclusion criteria were excluded (n = 24, 23, 24 in (a), n = 24, 23, 24 in (b), n = 24, 23, 24 in (c), n = 24, 23, 24 in (d)). Source data are provided as a Source Data file.

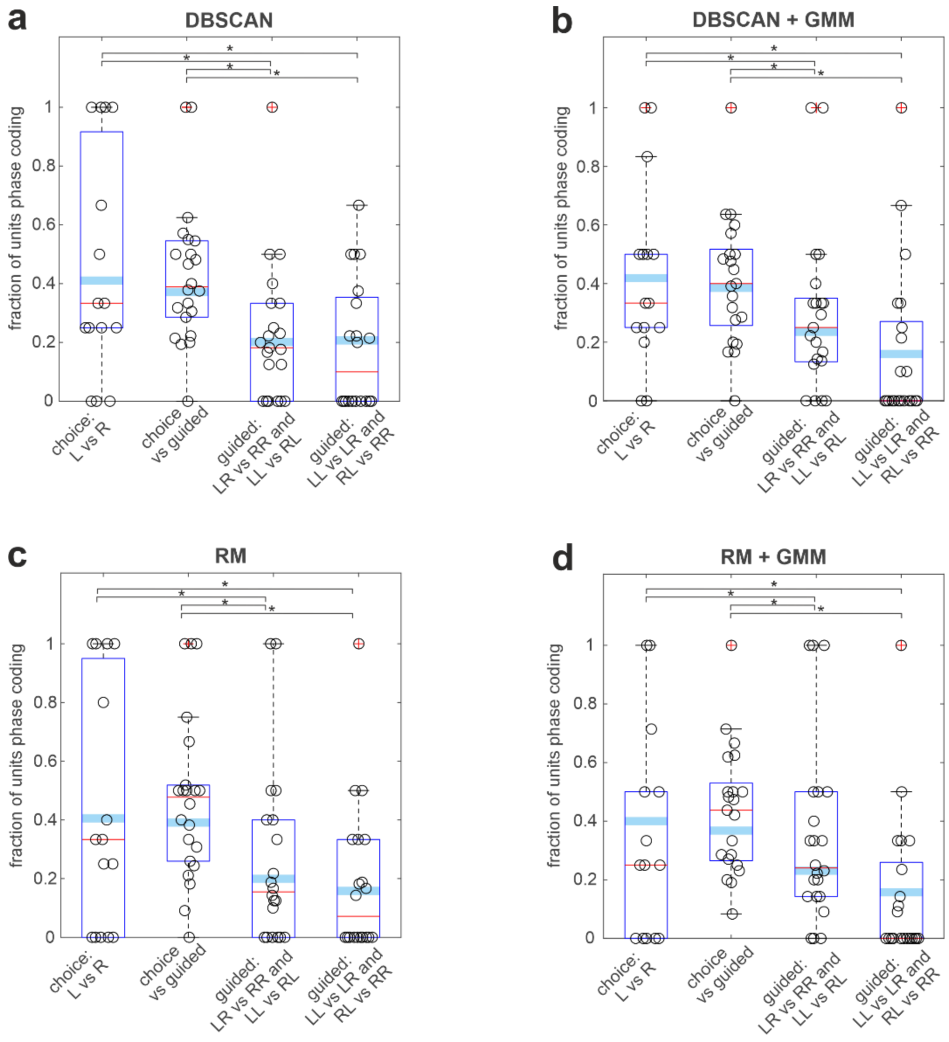

Supplementary Fig. 10 | Fraction of units changing preferred theta-phase for different sets of trial types. To probe the robustness of the results reported in Fig. 5d we performed the same analysis on sets of place fields identified with alternative techniques. From panel (a) to (d) we used place fields detected respectively by: a density-based spatial clustering algorithm (**DBSCAN**); the DBSCAN algorithm combined with a Gaussian mixture model (**DBSCAN + GMM**); thresholding the unit rate map (**RM**); the RM algorithm combined with a Gaussian mixture model (**RM + GMM**) (see Methods for discussion and description of the place field detection algorithms). In all four panels we tested significance with a generalized linear mixed-effects model to account for rat identity and recording session as covariates. We modeled if a unit changed firing phase (binary 0/1 variable and logit link function) within the same place field according to the trial types. The resultant statistics was (a) global F-statistics $F(4,613) = 14.4$, $p = 3.3\ 10^{-11}$; contrast tests between specific condition/bars (I vs II) $F(1,613) = 0.2$, $p = 0.6$; (I vs III) $F(1,613) = 6.9$, $p = 8.6\ 10^{-3}$; (I vs IV) $F(1,613) = 5.6$, $p = 1.8\ 10^{-2}$; (II vs III) $F(1,613) = 12.8$, $p = 3.7\ 10^{-4}$; (II vs IV) $F(1,613) = 8.5$, $p = 3.6\ 10^{-3}$; (III vs IV) $F(1,613) = 0.0$, $p = 0.9$; (b) global F-statistics $F(4,641) = 10.2$, $p = 4.9\ 10^{-8}$; contrast tests between specific condition/bars (I vs II) $F(1,641) = 0.2$, $p = 0.6$; (I vs III) $F(1,641) = 5.6$, $p = 1.8\ 10^{-2}$; (I vs IV) $F(1,641) = 10.7$, $p = 1.2\ 10^{-3}$; (II vs III) $F(1,641) = 9.8$, $p = 1.8\ 10^{-3}$; (II vs IV) $F(1,641) = 16.4$, $p = 5.8\ 10^{-5}$; (III vs IV) $F(1,641) = 1.9$, $p = 0.2$; (c) global F-

statistics $F(4,597) = 9.3$, $p = 2.9\ 10^{-7}$; contrast tests between specific condition/bars (I vs II) $F(1, 597) = 0.1$, $p = 0.7$; (I vs III) $F(1, 597) = 7.2$, $p = 7.7\ 10^{-3}$; (I vs IV) $F(1, 597) = 9.5$, $p = 2.1\ 10^{-3}$; (II vs III) $F(1, 597) = 14.8$, $p = 1.3\ 10^{-4}$; (II vs IV) $F(1, 597) = 16.6$, $p = 5.1\ 10^{-5}$; (III vs IV) $F(1, 597) = 0.6$, $p = 0.5$; (d) global F-statistics $F(4,631) = 9.3$, $p = 2.7\ 10^{-7}$; contrast tests between specific condition/bars (I vs II) $F(1, 631) = 0.2$, $p = 0.7$; (I vs III) $F(1, 631) = 4.3$, $p = 3.9\ 10^{-2}$; (I vs IV) $F(1, 631) = 9.1$, $p = 2.7\ 10^{-3}$; (II vs III) $F(1, 631) = 8.1$, $p = 4.7\ 10^{-3}$; (II vs IV) $F(1, 631) = 15.0$, $p = 1.2\ 10^{-4}$; (III vs IV) $F(1, 631) = 2.0$, $p = 0.2$. Panel (b) is the same as Fig. 5d and reported here for comparison. Data from 4 sessions of 6 rats, sessions where no units met the inclusion criteria were excluded (n = 15, 22, 22, 20 in (a), n = 15, 21, 21, 21 in (b), n = 15, 22, 20, 20 in (c), n = 14, 21, 22, 22 in (d)). Source data are provided as a Source Data file.

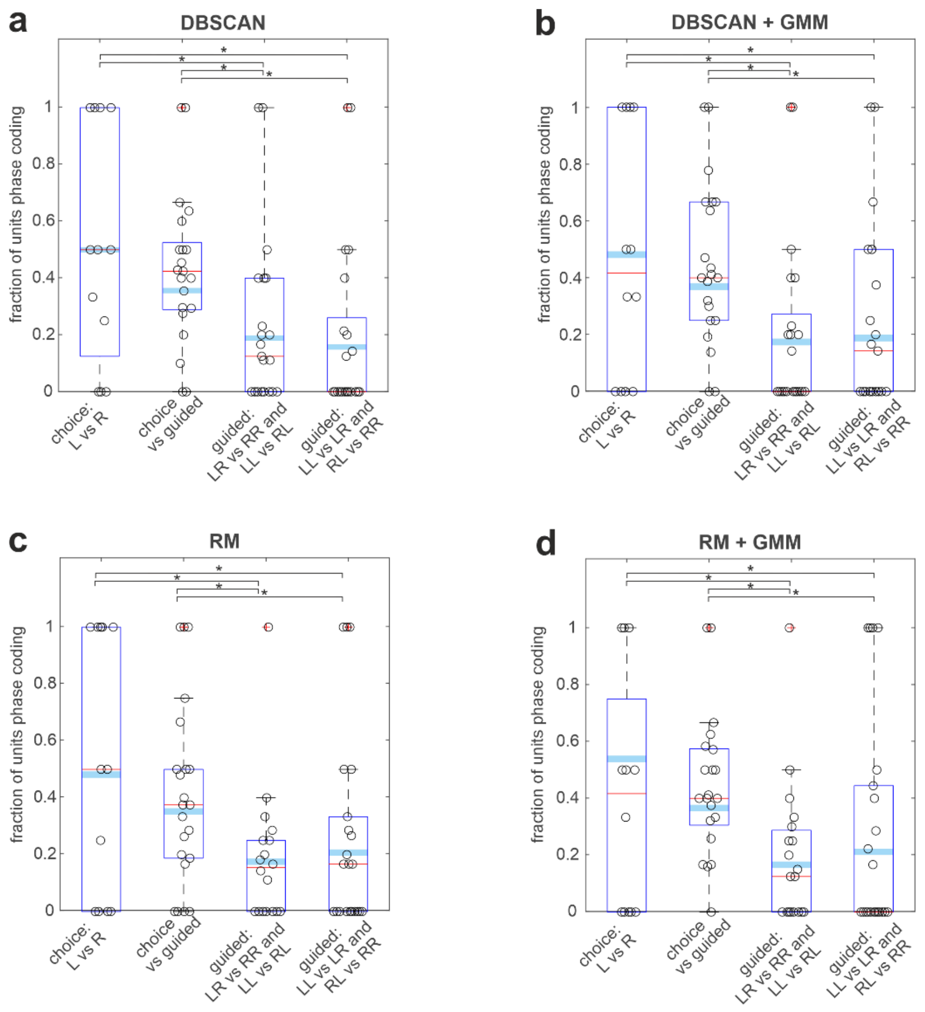

Supplementary Fig. 11 | Fraction of units changing preferred theta-phase for different sets of trial types (only high theta power and no SWR included). Same as Fig. 5d and Supplementary Fig. 10 but with analyses performed on spikes fired in epochs of high theta-power and outside of SWR epochs. In the four panels the resultant test statistics was (a) global F-statistics F(4,514) = 10.0, p = 8.2 10^{-8}; contrast tests between specific condition/bars (I vs II) F(1,514) = 2.2, p = 0.1; (I vs III) F(1,514) = 11.0, p = 1.0 10^{-3}; (I vs IV) F(1,514) = 12.5, p = 4.5 10^{-4}; (II vs III) F(1,514) = 11.5, p = 7.6 10^{-4}; (II vs IV) F(1,514) = 12.6, p = 4.2 10^{-4}; (III vs IV) F(1,514) = 0.3, p = 0.6; (b) global F-statistics F(4,515) = 8.2, p = 2.0 10^{-6}; contrast tests between specific condition/bars (I vs II) F(1,515) = 1.5, p = 0.2; (I vs III) F(1,515) = 11.1, p = 9.4 10^{-4}; (I vs IV) F(1,515) = 8.9, p = 3.0 10^{-3}; (II vs III) F(1,515) = 14.0, p = 2.0 10^{-4}; (II vs IV) F(1,515) = 9.6, p = 2.0 10^{-3}; (III vs IV) F(1,515) = 0.1, p = 0.7; (c) global F-statistics F(4,472) = 7.7, p = 5.3 10^{-6}; contrast tests between specific condition/bars (I vs II) F(1,472) = 0.2, p = 0.1; (I vs III) F(1,472) = 10.7, p = 1.1 10^{-3}; (I vs IV) F(1,472) = 7.5, p = 6.5 10^{-3}; (II vs III) F(1,472) = 10.2, p = 1.5 10^{-3}; (II vs IV) F(1,472) = 5.3, p = 2.1 10^{-2}; (III vs IV) F(1,472) = 0.4, p = 0.5; (d) global F-statistics F(4,507) = 11.7, p = 4.2 10^{-9}; contrast tests between specific condition/bars (I vs II) F(1,507) = 2.6, p = 0.1; (I vs III) F(1,507) = 13.4, p = 2.8 10^{-4}; (I vs IV) F(1,507) = 9.5, p = 2.1 10^{-3}; (II vs III) F(1,507) = 13.7, p = 2.3 10^{-4}; (II vs IV) F(1,507) = 7.6, p = 6.0 10^{-3}; (III vs IV) F(1,507) = 0.5, p = 0.5. Data from 4 sessions of 6 rats, sessions where no units met the inclusion criteria were excluded (n = 12, 21, 21, 21 in (a), n = 12, 21, 21, 21 in

(b), n = 15, 22, 18, 22 in (c), n = 12, 21, 19, 22 in (d)). This control confirmed that excluding spikes fired at low theta power and during SWR did not change the observed modulation in theta phase according to the trial type of some CA1 units. Source data are provided as a Source Data file.

Parameter	Value
$\boldsymbol{C}$	250 pF
$\boldsymbol{g_L}$	10 nS
$\boldsymbol{E_L}$	-58 mV
$\Delta_{\boldsymbol{T}}$	2 mV
$\boldsymbol{V_T}$	-50 mV
$\boldsymbol{E_e}$	0 mV
$\boldsymbol{E_i}$	-75 mV
$\boldsymbol{\tau_w}$	120 ms
$\boldsymbol{a}$	2 nS
$\boldsymbol{b}$	0.1 nA
$\boldsymbol{V_r}$	-46 mV
$\boldsymbol{V_s}$	20 mV

Supplementary Table 1 | Parameters of the adaptive exponential integrate-and-fire model. Parameters used to produce the results presented in **Fig. 6f**.

Supplementary References

1. Russo, E. & Durstewitz, D. Cell assemblies at multiple time scales with arbitrary lag distributions. eLife 6:e19428, 1–39 (2017).

2. Wirtshafter, H. S. & Wilson, M. A. Differences in reward biased spatial representations in the lateral septum and hippocampus. eLife 9, e55252 (2020).
