# Supplementary material for: Integration of rate and phase codes by hippocampal cell-assemblies supports flexible encoding of spatiotemporal context
Source: Nat Commun. 2024 Oct 22;15:8880. doi: 10.1038/s41467-024-52988-x (PMC11496817; doi:10.1038/s41467-024-52988-x)
Supplement: Supplementary file 3 — Reporting Summary [file 41467_2024_52988_MOESM3_ESM.pdf]

nature portfolio

Corresponding author(s): Eleonora Russo

Last updated by author(s): Apr 12, 2024

Reporting Summary

Nature Portfolio wishes to improve the reproducibility of the work that we publish. This form provides structure for consistency and transparency in reporting. For further information on Nature Portfolio policies, see our Editorial Policies and the Editorial Policy Checklist.

Statistics

For all statistical analyses, confirm that the following items are present in the figure legend, table legend, main text, or Methods section.

n/a	Confirmed	
☐	☒	The exact sample size (n) for each experimental group/condition, given as a discrete number and unit of measurement
☐	☒	A statement on whether measurements were taken from distinct samples or whether the same sample was measured repeatedly
☐	☒	The statistical test(s) used AND whether they are one- or two-sided<br>*Only common tests should be described solely by name; describe more complex techniques in the Methods section.*
☐	☒	A description of all covariates tested
☐	☒	A description of any assumptions or corrections, such as tests of normality and adjustment for multiple comparisons
☐	☒	A full description of the statistical parameters including central tendency (e.g. means) or other basic estimates (e.g. regression coefficient) AND variation (e.g. standard deviation) or associated estimates of uncertainty (e.g. confidence intervals)
☐	☒	For null hypothesis testing, the test statistic (e.g. F, t, r) with confidence intervals, effect sizes, degrees of freedom and P value noted<br>*Give P values as exact values whenever suitable.*
☒	☐	For Bayesian analysis, information on the choice of priors and Markov chain Monte Carlo settings
☐	☒	For hierarchical and complex designs, identification of the appropriate level for tests and full reporting of outcomes
☒	☐	Estimates of effect sizes (e.g. Cohen's d, Pearson's r), indicating how they were calculated

Our web collection on statistics for biologists contains articles on many of the points above.

Software and code

Policy information about availability of computer code

Data collection	Cheetah 5.1 (Neuralynx, MT)
Data analysis	Matlab2018a, Sleepwalker Matlab toolbox, Circular Statistics Matlab Toolbox. Code for cell assembly detection at multiple timescales CAD and CADopti available at https://github.com/DurstewitzLab/Cell-Assembly-Detection and https://github.com/DurstewitzLab/CADopti, respectively.

For manuscripts utilizing custom algorithms or software that are central to the research but not yet described in published literature, software must be made available to editors and reviewers. We strongly encourage code deposition in a community repository (e.g. GitHub). See the Nature Portfolio guidelines for submitting code & software for further information.

Data

Policy information about availability of data

All manuscripts must include a data availability statement. This statement should provide the following information, where applicable:

- Accession codes, unique identifiers, or web links for publicly available datasets
- A description of any restrictions on data availability
- For clinical datasets or third party data, please ensure that the statement adheres to our policy

The data generated for this study have been deposited in the open access repository OSF under the identifier DOI 10.17605/OSF.IO/PESKV accessible at https://osf.io/peskv/.

Research involving human participants, their data, or biological material

Policy information about studies with human participants or human data. See also policy information about sex, gender (identity/presentation), and sexual orientation and race, ethnicity and racism.

Reporting on sex and gender	n/a
Reporting on race, ethnicity, or other socially relevant groupings	n/a
Population characteristics	n/a
Recruitment	n/a
Ethics oversight	n/a

Note that full information on the approval of the study protocol must also be provided in the manuscript.

Field-specific reporting

Please select the one below that is the best fit for your research. If you are not sure, read the appropriate sections before making your selection.

☒ Life sciences ☐ Behavioural & social sciences ☐ Ecological, evolutionary & environmental sciences

For a reference copy of the document with all sections, see nature.com/documents/nr-reporting-summary-flat.pdf

Life sciences study design

All studies must disclose on these points even when the disclosure is negative.

Sample size	The UK Home Office Project Licence covering the procedures used here requires power calculations, as did the UKRI (BBSRC) funders of the work. Power calculations were performed using GPower software.
Data exclusions	To select putative place cells we restrict to units with firing rate between 0.2 Hz and 4 Hz and with spatial information above 0.5 bit/s.
Replication	The study examined a novel phenomenon. The study design did not contain generally full replication cohorts, however, analyis were designed to test the presence of the described phenomenon across animals. During the first round of revision, we were asked by reviewers to include more data to our analyses. In the revised manuscript, we added data from an additional rat and from two additional sessions for each rat of the cohort. All results of the analyses were confirmed by the new data.
Randomization	*Describe how samples/organisms/participants were allocated into experimental groups. If allocation was not random, describe how covariates were controlled OR if this is not relevant to your study, explain why.*
Blinding	blinding was not possible as all animals received the same treatment.

Reporting for specific materials, systems and methods

We require information from authors about some types of materials, experimental systems and methods used in many studies. Here, indicate whether each material, system or method listed is relevant to your study. If you are not sure if a list item applies to your research, read the appropriate section before selecting a response.

Materials & experimental systems

n/a	Involved in the study	
☒	☐	Antibodies
☒	☐	Eukaryotic cell lines
☒	☐	Palaeontology and archaeology
☐	☒	Animals and other organisms
☒	☐	Clinical data
☒	☐	Dual use research of concern
☒	☐	Plants

Methods

n/a	Involved in the study	
☒	☐	ChIP-seq
☒	☐	Flow cytometry
☒	☐	MRI-based neuroimaging

Animals and other research organisms

Policy information about studies involving animals; ARRIVE guidelines recommended for reporting animal research, and Sex and Gender in Research

Laboratory animals	Rat, Long Evans, adult males (300-500g), 9-16 weeks, supplied by Harlan UK
Wild animals	n/a
Reporting on sex	Information on the sex of the laboratory animals (male) is indicated in the manuscript. We expect the reported findings to apply equally to both genders.
Field-collected samples	n/a
Ethics oversight	All procedures were conducted in accordance with the UK Animals (Scientific Procedures) Act 1986 and approved by the University of Bristol Animal Welfare and Ethical Review Board.

Note that full information on the approval of the study protocol must also be provided in the manuscript.

Plants

Seed stocks	n/a
Novel plant genotypes	n/a
Authentication	n/a
